# Control Systems and Electronic Instrumentation Applied to Autonomy in Wheelchair Mobility: The State of the Art

**DOI:** 10.3390/s20216326

**Published:** 2020-11-06

**Authors:** Mauro Callejas-Cuervo, Aura Ximena González-Cely, Teodiano Bastos-Filho

**Affiliations:** 1 Grupo de Investigación en Software, Universidad Pedagógica y Tecnológica de Colombia, Avenida Central del Norte 39-115, Tunja 150003, Boyacá, Colombia; aura.gonzalez@uptc.edu.co; 2 Postgraduate Program in Electrical Engineering, Federal University of Espírito Santo, Av. Fernando Ferrari, 514-Goiabeiras, Vitória, Espírito Santo 29075-910, Brazil; teodiano.bastos@ufes.br

**Keywords:** automatic wheelchair, control, instrumentation, intelligent wheelchair

## Abstract

Automatic wheelchairs have evolved in terms of instrumentation and control, solving the mobility problems of people with physical disabilities. With this work it is intended to establish the background of the instrumentation and control methods of automatic wheelchairs and prototypes, as well as a classification in each category. To this end a search of specialised databases was carried out for articles published between 2012 and 2019. Out of these, 97 documents were selected based on the inclusion and exclusion criteria. The following categories were proposed for these articles: (a) wheelchair instrumentation and control methods, among which there are systems that implement micro-electromechanical sensors (MEMS), surface electromyography (sEMG), electrooculography (EOG), electroencephalography (EEG), and voice recognition systems; (b) wheelchair instrumentation, among which are found obstacle detection systems, artificial vision (image and video), as well as navigation systems (GPS and GSM). The results found in this review tend towards the use of EEG signals, head movements, voice commands, and algorithms to avoid obstacles. The most used techniques involve the use of a classic control and thresholding to move the wheelchair. In addition, the discussion was mainly based on the characteristics of the user and the types of control. To conclude, the articles exhibited the existing limitations and possible solutions in their designs, as well as informing the physically disabled community about the technological developments in this field.

## 1. Introduction

Technical aids allow the improvement of mobility in people with physical disabilities that affect their functional performance. In addition, it has to be considered how healthy or positive a person’s environment is and, thus, how their quality of life can be improved [1]. For this reason, a person is recognised within a sociocultural environment which is different from that of those who do not suffer from neuromusculoskeletal deficiencies and those related with mobility or others [2]. However, recent data show that there is still a field to explore and great concern regarding the numbers related with disability. For example, according to the World Health Organization (WHO), “Around 10% of the world population suffer from physical disabilities”, that is, about 650 million people [3]. From this percentage of the population who live with disabilities, 10% require the use of a wheelchair, but only a small number have access to them, and very few have access to the appropriate type of wheelchair [4]. Moreover, the “World Report on Disability” shows that 15% of the global population have some kind of disability, a number that is on the rise [3].

The people who have larger incomes have better access to manual or automatic wheelchairs than people with small incomes, including people over 65 years of age. On the other hand, there is evidence that disabled people often have limited living situations which can lead to them acquiring habits that damage their health [5]. In Colombia, according to the National Administrative Department of Statistics (DANE, by its acronym in Spanish) and the Ministry of Health and Social Protection (2012–2018), 2.9% of the total population have a disability of some kind, which represents 1,448,889 people. Out of this number, 34% of them exhibit a disability in the movement of the body, hands, arms or legs, representing a total of 496,522 people [5]. These figures call for a detailed study to be carried out regarding mobility and a solution to this problem.

A limitation in mobility significantly influences the lives of people as they cannot carry out their daily activities normally and require technical aids to improve their quality of life. In a report by the “Sala Situacional de Personas con Discapacidad en Colombia” (a registry of disabled persons in Colombia) of the Ministry of Health of Colombia [5], it is mentioned that there are 341,465 people that require assistance with mobility, representing 24% of the population with a disability in their upper or lower limbs. These figures lead to the conclusion that a wheelchair would be a solution to this problem, helping people with mobility issues in their upper or lower limbs, or both. Using one can significantly improve their quality of life.

On the other hand, the elements that make up a wheelchair depend on its type, be it manual or automatic. Nevertheless, manual wheelchairs require the strength of the user, which is why they are not recommended. A wheelchair should accommodate to the person so that they don’t have to use their energy to adapt to it. 

Those people who have a physical disability in their upper limbs require the assistance of another person to move. For this reason, automatic wheelchairs have been designed which allow the user to move around without a companion. These wheelchairs include a pair of motors for movement, as well as batteries to run the motors and a control system which varies depending on the needs of the person. If it is a commercial wheelchair, it often uses a joystick for movement. 

The control characteristics of wheelchairs is based on the current state of research and its implementation. Thus, the authors propose the following question, with the purpose of carrying out an adequate search of the scientific articles to review: Are the control systems that have been developed between 2012 and 2019 adequate for people with disabilities in their upper or lower limbs, or in both?

The control systems analysed in this document are divided into two types; those in which the sensors are located on the person (non-invasive) and those with the sensors on the wheelchair, depending on the needs of the user. On the other hand, the cost varies depending on the type of control system that is used. The objective of this investigation was to describe the state of the art in the period between 2012 and 2019, evaluating the different control systems for wheelchair movement. This analysis provides an understanding of the new parameters which will be implemented in future research in this field.

## 2. Methods

This state of the art was divided into four fundamental parts, which were: (1) a search of specialised databases in order to obtain information about the different types of controls of automatic wheelchairs which have been implemented between 2012 and 2019; (2) article inclusion/exclusion process; (3) data collection and information quality; (4) analysis and presentation of results of the articles reviewed.

### 2.1. Search of Specialised Databases

The databases that were used were IEEE Xplore, Research Gate, Springer, and Science Direct. The general terms included in the searches were: ALL (control AND wheelchair), obtaining 4688 results. Then, the search was refined with the equation: ALL ((*control AND wheelchair* AND (automatic OR intelligent))), obtaining articles that focused on the automatic control of wheelchairs using different techniques. Subsequently, a precise search was carried out focused on different categories in which specific terms such as the following were added (bci OR mems OR eog OR eeg OR emg OR hmi OR laser OR (‘voice recognition’)), delivering a total of 150 documents. The search equations were based on key terms, depending on the type of sensors to be implemented and the type of control developed in each application.

### 2.2. Selection and Inclusion Criteria

The selected articles were included based on the following criteria: (a) publication date: April 2012 to November 2019, (b) approach based on the control of automatic wheelchairs and on the instrumentation used on the chair or the user. In this study, 45 articles related with the physical design of the wheelchair were excluded as they solely focus on the electronic control of the wheelchairs. 

Using the previously mentioned inclusion and exclusion criteria, 150 articles and two theses—undergraduate and postgraduate—were found, of which 94 articles, two theses, and one book were included in this study for their respective analysis. The main reason for the exclusion of the remaining 53 articles was that, after reading the whole document, it was not possible to determine the type of control or the instrumentation implemented for the wheelchair to be automatic. 

The articles were selected considering as inclusion criteria the main characteristic of the design and implementation of the electronic controller, whether it was using sEMG, EEG, EOG, or a human-machine interface (HMI) or brain-computer interface (BCI), apart from sensors on the wheelchair, in order to evaluate the environment and make decisions, without taking into consideration the mechanical cost of the wheelchair. Works which only described simulations of the controller designed were excluded.

The 97 documents included in the study mentioned a detailed instrumentation of wheelchairs for their automatization, in addition to the design of basic and complex controllers, and the implementation of the controller on the wheelchair or the prototype. In total, 28 articles described the design of a controller, but it was not physically implemented; 12 articles are about the design of a controller implemented in a real wheelchair prototype, and 56 articles address the design and implementation of a controller in a finished wheelchair. Consequently, reading the articles selected provides information about the different control techniques and types of instrumentation in this area. Therefore, a division into two categories was made: sensor technology non-invasively placed on the user and wheelchair instrumentation.

### 2.3. Data Collection and Information Quality

The procedure to organise and classify the information was conducted starting from the results obtained with the search equations mentioned previously. Said information was extracted taking into account a limit of the article publications starting from 2012 up to 2019. Also, a compilation of 97 documents was carried out, which mentioned the control used on a prototype or wheelchair. Starting from the classification, the articles were read considering the abstract, as well as the results obtained and the conclusions. A synthesis of each one of them was made and it was determined if they applied to the present research. Finally, a classification was made based on what was more frequently found in the articles, dividing the information into the sensor part of the wheelchair, that is, which is not part of the chair itself, and the other division is the wheelchair’s instrumentation, which is not attached to the user, in contrast to the previous section which can be implemented non-invasively.

### 2.4. Synthesis, Analysis, and Result Presentation

In the results section, three fundamental divisions are presented:

- Participants in the characterization of controlled systems
- Instrumentation non-invasively placed on the user
- Instrumentation incorporated in the wheelchair

The first division covers the number of participants who corroborate the instrumented and controlled system of the completed wheelchair, prototype or robot.

The second division instrumentation non-invasively placed on the user with four sub-divisions: brain-computer interface, systems which implement MEMS, the third is surface electromyography, and electro-oculography, and lastly, other types of instrumentation.

Finally, the third division deals with instrumentation incorporated in the wheelchair, with the following sub-divisions: the first is obstacle detection; the second is artificial vision and finally, wheelchair navigation. Said topics are developed making special emphasis in the types of controls implemented in the wheelchair.

## 3. Results

**Figure 1:**
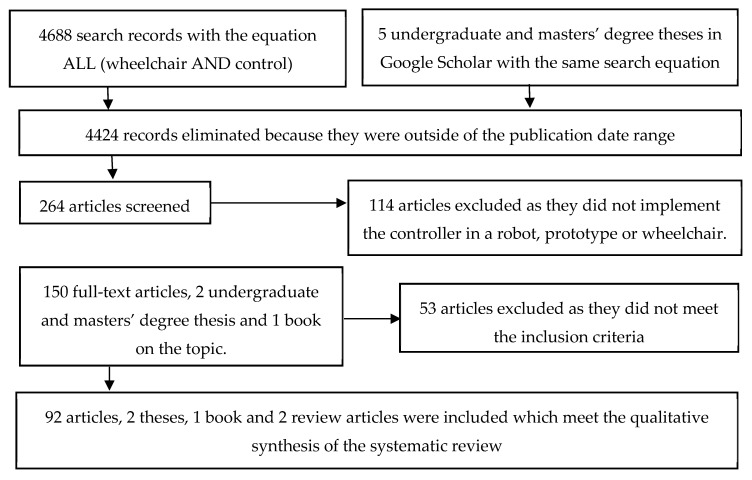
State-of-the-art phases of the control systems of automatic wheelchairs according to the PRISMA format.

The results analysed in the state of the art are related with the user through a graphic interface, sensor signals; study of the immediate environment, and safety with regard to collisions or falls when using the wheelchair. Touch screens, the use of graphic interfaces, applications, micro-controlled systems, among others, allow the user to move by only interacting with a human-machine interface or by generating a body signal which indicates the action the user wants to perform. The flow diagram which presents the information about the different phases of the state of the art are shown in Figure 1.

Before mentioning in detail each type of instrumentation and controller, the following Table 1 shows the input methods of the controllers in the articles reviewed.

Also, in Table 2, the control strategies used in the articles reviewed are mentioned in a general fashion.

### 3.1. Participation in the Characterization of Controlled Systems

Data was collected to find out the number of participants who were involved in the development of controllers in both divisions. This information is useful, given that in the development of an electronic controller the characteristics it should meet are evaluated. Therefore, the authors of the articles reviewed mention the need for the characterization of controllers with regard to specific participants.

From the 98 articles reviewed, those controllers which were tested in completed wheelchairs required 254 participants for their collective development and testing. Table 3 presents the number of participants for each classification division of the electronic controllers.

Most of the articles that tested the controllers with the help of participants indicate that the controller developed worked successfully. However, controllers that involve the use of BCI indicated that the concentration of the patient was required, as mentioned in Ng et al. [16] and Carrillo et al. [17]. Furthermore, the signals obtained through sEMG and EOG require specific characteristics of the participants. Regarding the navigation of the wheelchair, the authors mention that the versatility of the wheelchair is important, as well as avoiding the inconvenience of making a design for specific patients. 

On the other hand, the authors stand out because they are mainly Asian, given the boom in this continent with respect to automation and process control, in this case, in the health area. Nonetheless, a study was conducted in Uganda, proving that technical aids to mobility offer more possibilities in work and education contexts [3]. Below, the description of the literature selected for this information analysis is presented. The following section refers to sensors incorporated non-invasively on the wheelchair user and the types of controllers developed. 

### 3.2. Instrumentation Non-Invasively Placed on the User 

The control systems mentioned in this section are based on sensors which are placed on the user in a non-invasive manner, and which are closely related in that they implement a human-machine interface (HMI). Within the interfaces, specific systems are connected, which take body signals and turn them into control signals for the operation of the wheelchair, as electrical impulses. For example, the following are used: electroencephalography, micro-electromechanical sensors in order to capture head and hand movements, electrical impulses generated by muscular movement, eye movement or electro-oculography and, finally, other types of instrumentation. 

#### 3.2.1. Brain-Computer Interface (BCI)

A BCI is an interaction between the brain of the user and a computer or microcontroller system, which executes control actions over the wheelchair. For this reason, the use of electrical impulses is indispensable in this field, making EEG the correct method of using BCI. From the review in this area, 20 articles are found that use EEG, in the knowledge that these systems require additional instrumentation for their functioning, such as the systems of communication and instrumentation needed for the distance sensors for the safety of the wheelchair. Also, two articles involved the use of EOG and EEG, which are two forms of wheelchair control. Consequently, 12 articles implement EEG and systems of communication, navigation, obstacle detection, functional magnetic resonance imaging (fMRI), and MEMS as a way of controlling wheelchairs. Table 4 indicates the instrumentation and control strategies implemented in each article that uses BCI. 

Going deeper into these implemented control systems, in Widyotriatmo et al. [6] a robotic system is developed that uses EEG signals to control the motors. It also incorporates two additional systems to follow walls and avoid obstacles. It is a hybrid system that has a camera as a localization system, but the main control lies in the interface between the EEG signals and the wheelchair. A dual system is implemented by Long et al. [7] in which EEG signals are captured and it takes images in response to the eye blinking, in order to control the direction and speed of the wheelchair. The steering of the wheelchair is carried out through a graphic user interface (GUI). The control systems acquire motor imagery signals and pattern extraction. A BCI is presented by Dabosmita et al. [8] that extracts brain signals in order to recognize the intention of the user regarding the movement of the wheelchair. It includes a GUI made in LabVIEW^TM^. It uses Arduino and a Bluetooth module for the functioning of the wheelchair prototype. The EEG signals are filtered and processed through a graphic interface to be later sent as control signals to the prototype. A control system is presented by Kobayashi et al. [9] which describes a method of fractal emotion analysis for the movement of the wheelchair, with the use of specific emotions, such as anger, sadness and pleasure. With these emotions as input signals to the controller, the respective movements are made by the wheelchair. 

These controllers do not involve the use of other types of sensors in the wheelchairs instrumentation or a robotic arm for their movement. Jayabhavani et al. [10], develop an interface that captures EEG signals received by mobile phone and executes the actions of control, sending the signals wirelessly. A camera that detects obstacles is located on the wheelchair as well as a wi-fi module to receive the control actions. A characteristic of this type of controller is that it is run in a mobile phone. A system is implemented by Zgallai et al. [11] that uses EEG and EOG for wheelchair movement. The EEG system has 16 channels for receiving signals and ultrasonic sensors to detect obstacles; it also has a GPS module to locate the wheelchair. The signals are processed by a neural network in order to characterize them and a microcontroller receives this characterization as input for the execution of the controller to drive the wheelchair. This system is a hybrid since it has a BCI and a navigation system with the GPS module for the spatial location of the wheelchair. A prototype is developed by Kim et al. [12], based on EEG which uses five classes of motor imagery for the movement of the wheelchair in 5 directions. Processing is carried out by Simulink and MATLAB^®^ scripts. The system requires a network module for communication between the wheelchair and the BCI. This is a BCI system which does not involve another type of sensor for control. An interface is developed by Reshmi y Amal [13] for controlling a prototype wheelchair through the use of five classes of motor imagery. The classification of the signals was done by implementing neural networks made in MATLAB^®^. Concentration is required for the use of this type of control since inattention can execute erroneous control actions. The system was developed by testing it on 50 people to distinguish behaviors and to fulfill the control objective. A BCI interface is developed by Jang et al. [14] to drive a wheelchair prototype that must fulfill specific tasks. The user cannot make any body movements; therefore, the prototype is a kind of technical aid. The prototype is equipped with a 2D gyroscope and a camera. It is controlled by the person blinking their eyes and signals are acquired via EEG. This type of system does not require other instrumentation as the prototype meets the desired characteristics. An automatic wheelchair is designed by Huang et al. [15] which is operated through EEG, as well as using a GPS module to establish routes and reach its destination. The routes are visualised using the LabVIEW^TM^ interface and it includes sensors for obstacle detection. The user can change the orientation of the wheelchair via EEG. When the wheelchair is being operated in real time, in which the user imagines a direction of movement, such as right or left, upon recognising these signals, which it was taught to do previously, the wheelchair is directed in that direction.

In Ng et al. [16] a BCI is implemented through steady state visually evoked potentials (SSVEP), which are signals that respond to visual stimuli at determined frequencies. It has an interface in order to determine the place at which the user wants to arrive. It is a hybrid system, given that it involves a BCI and an obstacle detection system. It also has sensors to avoid obstacles. The prototype was tested with 62 people in order to evaluate the wheelchair control. A BCI is designed by Carrillo to control a wheelchair using SSVEP. The orientation of the wheelchair depends on the directions in which the user wants to move. It requires a certain level of concentration to operate this system, as not everyone who tested the system was able to control the wheelchair. It does not come with another means of control [17]. A wheelchair is designed and built by Lasluisa [18] that operates with EEG, using an interface elaborated in LabVIEW^TM^ and the controller in Arduino. It has three operation modes: through gestures, cognitive mode and gyroscope mode. It has sensors for obstacle detection. It is a hybrid system as it involves a BCI and a navigation system. A BCI is designed by Pinos et al. [19] that takes signals from cognitive responses. The system used “mu” rhythms, which are the result of motor responses. The user must be trained to use this interface since it requires a certain level of concentration; the interface is developed in LabVIEW^TM^ and does not require other instrumentation for the control of the wheelchair. A control strategy is implemented by Chen et al. [20], using EEG signals, which manipulates a robotic arm incorporated into the wheelchair. The wheelchair has eight photoelectric sensors which detect movement by detecting the movement of arms and legs. In addition, the wheelchair has an implicit robotic arm for object manipulation.

A system is implemented by Su et al. [21], which acquires the EEG signals and transmits them via Bluetooth to MATLAB^®^ and a GUI collects the data and compares the images of the acquired EEG signals with images previously stored in the computer. The data collection generates a data rate, which is processed and converted into the chair’s control actions by means of thresholds. A method of task classification using somatosensory evoked potentials is developed by Kim and Lee, for the control of a prototype wheelchair by BCI. It uses the Fourier transform, filtering and spatial patterns for data extraction. It does not require any other type of instrumentation; therefore, it is a system that only involves a BCI [22]. A prototype that uses a Raspicam sensor for obstacle detection, an IMU and a speed control in steep areas are integrated with bio-signals to control the chair which is described in Turnip et al. [23]. The images taken by the Raspicam are binary and a classification algorithm is used to detect obstacles, in addition to the use of EEG signals to control the wheelchair. The motors are controlled by Raspberry Pi. When processing the image captured on Raspicam, the EEG signals are also processed in Raspberry Pi. In addition, the chair has ultrasonic sensors integrated with the Raspicam sensor. The system is a hybrid as it involves a BCI, an obstacle detection system and a navigation system. In Shahin et al. [24] an HMI is implemented to control the wheelchair through EEG with the Emotiv Epoc device, which was non-invasively placed on the head of the user. The signals were electrically coded and were sent wirelessly to a computer to be processed and translated into the control signals of the wheelchair. Two control systems are proposed: monomodal and multimodal. In the monomodal control the chair is controlled through facial expressions and the multimodal control takes advantage of all the head movements, the facial expressions and thoughts, so that users with different types of disabilities can use the chair. The system presumes that all the inbound signals of the Emotiv Epoc have the same weight and are combined using the simple sum-rule fusion technique. A GUI is implemented to manipulate both types of controls. A multimodal system was developed by Borges et al. [25] which has a virtual environment which trains users of electric-powered wheelchairs to handle them through sEMG, EEG and EOG signals. The algorithm has its basis on the use of thresholds and sEMG signals avoid false peak detections. Additionally, the use of neural networks is involved in order to recognise blinking. EEG signals use SSVEP. Finally, ref. [26] designs a wall tracking control for the movement of a wheelchair. The control is based on Lyapunov’s method and the control is of an adaptive type. This type of control is carried out by means of a BCI. In this case, a kinematic model is established for the wheelchair where linear and angular speed are taken into account.

#### 3.2.2. Systems Which Implement Micro-Electromechanical Sensors (MEMS)

Micro-electromechanical sensors are placed on the body or on the wheelchair in both categories. Table 5 mentions the articles which carry out said implementation in a non-invasive way on certain parts of the body and those that use MEMS in the instrumentation of the wheelchair. However, in this sub-division the ones that stand out are those whose main function lies in non-invasive implementation on the body for the control of the wheelchair. 

##### Controllers Based on Hand Movements

By looking at the MEMS sensors used for automatic wheelchair control, a hand movement control system using MEMS is designed by Lu in [52]. The system is conditioned to obtain the control signals. By recognising the gesture using Bayes’ method, a control command is sent to the wheelchair that translates a movement or change in speed of the chair. The control of the wheelchair is based on the linear and rotational speed of the wheelchair. The movement of the chair is right, left, back, forward, and stop. A control system is developed by Ruzaij et.al, using MEMS sensors in the user’s hand. It has a PID control developed in MATLAB^®^ and a microcontroller to drive the prototype [53]. An automated system for controlling chair rotation through head and hand movements is described in. It uses ADXL330 sensors with low power voltage signals. It also has an obstacle detection system [54]. A control system is developed by Kaur and Vashist, using MEMS sensors in the user’s hand. It has a PID control developed in MATLAB^®^ and a microcontroller to drive the prototype. It has no other type of instrumentation, so it is typical of this classification in MEMS sensors [55]. On the other hand, a control is developed by Postolache et al. [56], that monitors the physiological stress parameters and the movement activity of the wheelchair. A mobile application is made, in addition to the sensors imposed on a fibre which can be washed and reused.

##### Controllers Based on Head Movements

In Chen et al. [57] a system is developed that collects signals from tilt sensors located on the user’s head which are amplified and processed. They are then processed by a controller and drive the wheelchair in all four directions. A head movement recognition system is proposed by Dobrea et al. [58] to control the wheelchair. This system is proposed to be used by people with quadriplegia. The system’s sensors are of the capacitive type and are located in a tie. The system receives and processes signals through feature extraction, signal classification and uses artificial intelligence in this classification.

A communication system for operating the wheelchair is also designed in Kader et al. [59]. The system uses tilt sensors located on the user’s head, as well as wireless modules for the transmission and reception of information. The system distinguishes the distance of nearby obstacles and has ranges to operate the chair if it detects them. The range of distances is determined by the designer of the algorithm. In Dey et al. [60] a control system is implemented by means of head movement for the mobility of the chair. The system is initialised and must identify a specific movement to drive the wheelchair. A control system is developed by Marins et al. [61], that captures head movements by means of an IMU and a microcontroller takes these signals to drive the motors according to the information received. The classification of the data is done using MATLAB^®^. An interface is implemented by Gomes et al. [62] that uses inertial sensors placed on the head to steer the wheelchair, in addition to controlling the speed of the wheelchair. The chair has infrared sensors for obstacle detection, making the system a hybrid, as it implements MEMS sensors and an obstacle detection system. Moreover, in Ruzaij et al. [37] a voice recognition control system is described which can be used for people over 65, people with quadriplegia or other types of physical disabilities. It has MEMS inertial sensors located on the person’s head for wheelchair mobility. The system is a hybrid as it has MEMS sensors and a navigation system. 

The references in this section involve the movement of the head, but not the use of MEMS sensors. A system is designed by Yoda et al. [27] which uses head movements to control the wheelchair, and a graphic interface is used to interact with the user. Within the instrumentation of the chair there is a stereo camera and, a system that processes data. This system uses an algorithm to detect the orientation of the head and the estimated angle. The information is processed through a state machine. Manta et al. [28] propose a command interface based on the movement of the head for the control of the wheelchair, at the same time that it recognises the speed of the execution of said movements to be drawn in a 3D plan and avoid collisions. Rohmer et al. [63] present an automatic control for wheelchairs that involves sEMG signals along with a laser sensor which projects on the ground the direction the wheelchair should go. It also has a Kinect sensor which determines if the chair can complete that action. sEMG signals validate the route and a PID control is used in order to activate the motors. 

#### 3.2.3. Surface Electromyography (sEMG) and Electro-Oculography (EOG)

Muscular impulses are analysed through a technique denominated sEMG and ocular movements through EOG. These techniques can be useful to carry out actions which the user requires for the control of a wheelchair. Table 6 indicates the instrumentation and control strategies implemented in each article which uses controllers based on signals or body movements.

##### Controllers Which Implement EOG

A control technique is proposed by Challagundla et al. [66] using low intensity infrared (IR) radiation to the eyes and the voltage generated by the sensors depends on the movement of the eyelid. The opening or closing of the eye determines the forward or backward movements of the wheelchair, while lateral movements translate the direction in which the wheelchair should go. The eye gesture control was designed by Pingali et al. [67] and implemented using EOG. The design has an integrated control module that processes the bioelectric signals generated by eye movements. In addition, a PID controller is implemented to stabilise the system. A proof of concept was carried out by verifying the movement of the chair with the movement of the eyeball. A control based on eye movement is designed by Pangestu et al. [29] A neural network is implemented to learn to recognise the state or characteristics of the eye based on a bank of photos of eyes in different open and closed positions. The Viola-Jones algorithm for face and eye detection is implemented. The method is effective and does not require any other type of instrumentation for its execution. A learning mechanism is described by Rajesh and Mantur [30] that makes the system use EOG to control the chair. A training method is used for movement recognition using neural networks that differentiates the eye’s own characteristics, which are recorded with a camera. With regard to Wanluk et al. [31], the system describes 4 modules which include an image processing module, a wheelchair control module, another one for the monitoring of messages, and a last one for the control of household appliances. The wheelchair moves in the direction that the eye moves in. PLC offers more reliability, processing power and easy access to the different Input/Output functions. First, the joystick or tactile port are chosen, so as to determine the input data. Then, there is a choice to be made between directional or rotational control, and a PWM is generated to control the motors.

##### Controllers Which Implement sEMG

In addition, a joystick control system enhanced with sEMGs placed on the shoulder is implemented in Hardiansyah et al. [68]. These signals are conditioned so that they can operate the wheelchair. The system does not require any other type of instrumentation for the execution of the controller. A control scheme specifically for use by persons with quadriplegia is presented in Jang et al. [69]. The muscles of the face are what are used, which generate control signals for the movement of the chair. It is a hybrid system, as it has an obstacle detection system. Furthermore, Jang et al. [70] is the predecessor of Jang et al. [69]; as it describes the kinematic model and control method for the wheelchair through the use of a myoelectric computer interface (MCI) that controls the speed and direction of the chair. On the other hand, Küçükyildiz et al. [71] describes the control of a wheelchair using sEMG. The processing was done in MATLAB^®^ and has a Kinect device for the navigation of the chair; depth samples are processed and obstacles are avoided.

The EMG’s characteristics not only serve to control the wheelchair, but also indicate characteristics of the patient when performing physical effort, as indicated in Fortune et al. [72], which uses inertial sensors on the chest and shoulders to verify the physical effort made by the patient when moving in a wheelchair. Neural networks are used to analyse the signals from the sensors. Another feature is the development of a virtual wheelchair prototype controller through sEMG for those who have only operated the chair via joystick. The development is done by means of a virtual simulator and a server for data transmission [73].

#### 3.2.4. Other Types of Instrumentation 

A human-machine interface uses elements that can interact with the user, such as tactile screens, voice recognition systems, among others. In this sub-division, the HMI implemented for the automatic movement of the wheelchair is delved into. Table 7 indicates the instrumentation and control strategies implemented in each article that uses controllers based on other types of instrumentation.

For example, a system to go up and down the stairs with a wheelchair is designed by Tokhi et al. [90]. The design of the system requires three main actuators, a pair of motors for the wheels, and another one for the position of the seat. The control was carried out through fuzzy logic and allows the exchange of wheels. At the same time, Karpov et al. [38] designs a multimodal control for a wheelchair which can be used by any user. The user can enter a different signal for the movement of the chair, for example their voice, BCI, manual signals, or ocular movement, which converts it to a hybrid system, in which the wheelchair is instrumented in order to control the movements and has a camera for the detection of ocular movement. A semiotic model processes the sensorial data and plans the actions as a sequence of tasks for the control system. It uses neuronal networks for the BCI. At the same time, Boucha et al. [39] develops a control through the recognition of voice patterns and EEG. Control through EEG requires concentration and is carried out using Emotiv Epoc. Voice control is done in real time using the hidden Markov model (HMM). In addition, the control is conducted using relays: two circuits, one for the house, given that the system allows the control of household electronic devices, such as the turning on and off of lights and televisions; and one for the wheelchair, that is, to control it. A voice recognition system is implemented by Ruzaij and Poonguzhali with a tactile interface. The main advantage of this work is its low cost. A liquid crystal screen (LCS) alerts the user about excess of noise in a certain place in case the person cannot hear or understand the commands, and it also has ultrasonic sensors to detect obstacles [40]. 

A system is developed by Wang et al. [41] that changes the posture of the patient when sitting on the wheelchair, by the recognition of voice commands. The main objective of this project is the design and implementation of the control system of a voice operated wheelchair which can be adjusted into sitting, standing, and fully reclining position. An HMI is described by Gulpanich et al. [47] which wirelessly communicates, accessing through an application in a smartphone, for the convenience of the people who use it, as it regulates the direction and the speed of the wheelchair. There is control using a traditional joystick and the design of a Programmable Logical Controller (PLC) based on the integration of IoT (internet of things) for the interface and control of the wheelchair. A wireless multifunctional system is described by Makwana and Tandon which uses a tactile screen and is programmed through a microcontroller. The chair has ultrasonic sensors to avoid obstacles and a tactile screen which receives commands for the chair to operate [48]. In Umchid et al. [42] a voice recognition module compares the user’s voice with commands that were previously recorded. This system has ultrasonic sensors for the detection of obstacles. The system is controlled with a microcontroller which is the main unit of the wheelchair. It receives signals from the voice module and the ultrasonic sensors, and sends the control action to the relay module for the conditioning of the motors. Aktar et al. [43] present the design of a voice recognition system which identifies the commands received through a wi-fi module to control the wheelchair. The location of the chair can be obtained because it has a GPS which sends the information to a mobile application through Firebase, which is a platform for the development of web and mobile applications. This is a hybrid system because it relates an HMI with the detection of obstacles and a navigation system. At the same time, Aktar et al. [49] presents a system for people with physical disabilities which is controlled by a joystick. Clearesta et al. [82] implement a wheelchair control that regulates the speed and direction of the chair. It is an adaptive control that adds Lyapunov stability criteria. It uses a joystick as the input command. Matsuo and Barolli [83] describe the development of an omnidirectional wheelchair prototype, but it does not have an obstacle detection system and, therefore, it constantly collides with things. Finally, Cho et al. [97] describe a control for the posture of the wheelchair that has a pendular mechanism, as well as a detector of the user’s angle and posture.

### 3.3. Instrumentation Incorporated in the Wheelchair 

Instrumentation on the wheelchair involves a sensor which is placed on it for its mobility in open and closed spaces. This category is composed of three broad branches regarding instrumentation, which are: obstacle detection, artificial vision, and wheelchair navigation. This sub-division will be delved into based on the revision of the documents which discuss the topic of automatic wheelchair control. 

#### 3.3.1. Obstacle Detection

Table 8 shows the instrumentation and control strategies implemented in each article that uses controllers based on obstacle detection.

To begin, obstacle detection is vital for the full functioning of the wheelchair, thus, in this sub-division the systems whose main component is obstacle detection will be analysed in depth. Zhang et al. [74] describe a system that aids in going up stairs using the wheelchair and avoid collisions when the chair is being used in complex environments. The control strategy includes ultrasonic sensors and an input command, and the system is based on the virtual force field principle. It includes an encoder to measure the speed at which the chair moves and a joystick for its basic control. Sumida et al. [32] develop a navigation system which is based on the collection of data about its environment in order to be able to trace routes, using a GPS, which is safe and indicates to the person where they have to go. The system implements the Dijkstra algorithm, which finds routes which have the lowest cost among the nodes, which are assigned at a certain constant distance. Taniue et al. [75] present a system that detects barriers for wheelchairs, such as differences in the level or holes in the trajectory. The system remembers where the obstacle is and draws it on a digital map. Lee et al. [76] describe the development of a control with fuzzy logic for the monitoring of walls for a wheelchair that can avoid obstacles. The fuzzy logic control has 16 rules. Four ultrasonic sensors are placed on the wheelchair to send signals to the controller and be able to perform actions. The characteristics are designed based on the distance sensors. For their part, Natraj et al. [33] describe a real-time system with a laser camera which discerns obstacles at a certain distance. The image is processed and conic coefficients are used which measure the distance. Finally, Maatoug et al. [77] show a fuzzy logic control technique which uses multiple sensors as input signals. The objective is to track down the wheelchair using Kalman filters. A cinematic model of the wheelchair is determined and it independently controls the position and the orientation of the wheelchair.

#### 3.3.2. Artificial Vision 

In the health area, specifically in the case of technical aids, artificial vision has been implemented with the objective of capturing images of the environment and processing them, in order to help the user to move around a certain place, whether it is open or closed. The first part of this subsection refers to the digital image processing techniques with “Field Programmable Gate Array” (FPGA), and the second part with an image or video camera, and other sensors, such as Microsoft Kinect. Table 9 indicates the instrumentation and control strategies implemented in each article which uses controllers based on digital image processing.

##### Digital Image Processing Through FPGA

Delving into the topic, Zhao et al. [94] develop an object detection system by using a FPGA. The system implements an algorithm for the detection of key points in each video sample. A specific use of wheelchair navigation through FPGA is seen in Megalingam et al. [87] which implements a system based on FPGA that detects the floor of the user’s house and compares the squares of the floor, thus, calculating the movement the user has to make. MATLAB^®^ is involved in the implementation of the system. Each square is considered as an origin and destination. If a specific route is entered in the GUI, the wheelchair moves until it reaches the destination square. Obstacle detection is carried out by identifying objects and collecting images in a memory. Kathirvelan et al. [44] develop a control system for wheelchairs through LabVIEW^TM^ and FPGA which integrates ultrasonic sensors for obstacle detection. LabVIEW^TM^ is in charge of processing voice commands. The system is combined with an FPGA for the control and monitoring of the system modules. The main feature of the system is its low cost. 

##### Cameras or Optical Sensors with Different Characteristics

In Chang et al. [88] the user’s hands are recognised. The chair moves by means of sensors placed on the ceiling so that it can make the journey. Other methods, such as that used by Ahmad et al. [98] for the use of artificial vision in mobility using wheelchairs, describe a colour tracking technique. A system is developed by Wu et al. [95] where multi-sensorial data is used to track a companion. Maps are constructed and the person is searched for based on a laser range and inertial sensors. The recognition of the person is done by a Pan-Tilt-Zoom (PTZ) camera. The system has a physical wheelchair, the PTZ camera, an encoder sensor to measure the speed of the chair, a gyroscope, a touch panel and laser sensors for obstacle detection. A visual recognition controller for head movements using a digital signal processor (DSP) and a computer is designed and implemented in Jia et al. [64]. The wheelchair has six ultrasonic sensors, a joystick and a Webcam Pro. The Adaboost algorithm for face detection is implemented and runs in real time. A method is developed by Solea et al. [50] that translates the position of the head into a direction to control the wheelchair. It has a video camera, encoder, touch screen, data acquisition board (DAQ), power circuit and the wheelchair. This system implements a PID controller for the execution of the system and uses Kalman filters to be able to read the signals accurately. A method is implemented by Couceiro et al. [34] where optic flow vectors are used, placed in the visual field of the camera located on the legs of the user. The wheelchair follows the movement the leg indicates through images captured by the camera. The relative speed is calculated based on the processing of the image. Finally, Motokucho and Oda [78] develop a type of control that adapts to the environment by detecting objects and asking the user what kind of objects they are. A stereo camera is used for object recognition. Neural networks are implemented for this recognition. The control determines which object can be an obstacle to the chair and indicates the wheelchair’s movement intentions by means of an interface. 

#### 3.3.3. Wheelchair Navigation

Wheelchair navigation involves different techniques, in which communication systems are used for wheelchair mobility such as GPS modules, GSM, improvements in the use of joysticks, tracking control, use of touch screens, among others. Table 10 shows instrumentation and control strategies implemented in each article that uses controllers based on wheelchair navigation.

##### Linear and Hybrid Control

To begin with, Chen and Agrawal designed a system that follows a line by using the joystick in a closed-loop control. The training protocol is included in three groups. Control (CT), which receives no force feedback; AF, which uses assistive training when necessary; and RF with repelling forces. The system is implemented with a laser sensor, which follows a line that is located on the ground, it must be predefined for the wheelchair to follow that route and the joystick handles the tracking error [84]. On the other hand, a control is implemented by Sato et al. [85], in which several wheelchairs move due to the tracking of a companion. The system uses a map to estimate the position of the companion. In small spaces, the chairs are lined up behind the companion. A system was developed by Makwana et al. [89] which sends messages via GSM, the detection of obstacles and the monitoring of heart beats. The data collected from the monitoring is sent via WiFi so that an expert in the area is monitoring the user. In addition, the location of the person is sent by means of longitude and latitude. A robust control is implemented by Feng et al. [86] which takes into account the torque generated by the wheelchair and feeds it to the controller. This is a proportional-integral (PI) control designed in MATLAB^®^ and is simulated to observe the response of the controller. Park et al. [99] describe a basic control system based on the propulsion and rotation model of the wheelchair, and it also has a method of distributing the torque of the wheelchair. The control can be adjusted depending on the sensitivity coefficients of the joystick. A trackball joystick for wheelchair control, an LED matrix and a camera are implemented by Sreejith et al. [93]. The chair is guided by the flashing pattern, which is recognised by the camera installed on the wheelchair. An intelligent navigation system is proposed by Sivakumar et al. [51]. The system operates manually or automatically. When a voice command is recognized, the chair follows the route that has been pre-established. In addition, Chocoteco et al. [91] designed a system to ascend the stairs with a wheelchair using distance sensors. The posture and locomotion control is executed in LabVIEW^TM^ together with a PI and PD control. In Liang et al. [96], describe a system in which a wheelchair and a bed interact, as it has a reference point for the wheelchair to reach and the intelligent bed to approach so that the patient can be accommodated without the aid of a companion. 

##### Adaptative, Predictive and Intelligent Control

In Rabhi et al. [92] describes a modified design of the traditional joystick, which is based on the training of a neural network that is connected to the output of the joystick. The interface is made in LabVIEW^TM^. The neural network receives the input signal from the joystick and creates suitable signals for the motors. The signal has an acceleration and rotational movement reference. The user makes predefined paths to observe the error when training the network. On the other hand, Puanhvuan et al. [35] describe the control of a wheelchair through eye movements. It takes 5 s for the eye calibration, enabling the system. The system has an odometer, a laser scanner and a 3D camera. A navigation map is made for the house and the system can be controlled manually or automatically. A wheelchair navigation system is presented by Rhabi et al. [45] in environments with obstacles and exposed to random situations. A controller with fuzzy logic is created, which takes into account changes in the environment in which the wheelchair is located. A predictive model for the tracking controller for a wheelchair route is presented in Nguyen et al. [46]. The route has a linear treatment in which state variables are evaluated for the design of the controller. The control technique is a predictive model and its evaluation when implemented indicates that on circular routes the control responds correctly, however, on random routes, the control has a response time greater than 4 s. A communication system is developed by Tsunoda et al. [79] between an external environment and the wheelchair by means of visible light. An LED matrix and two cameras use bi-directional communication. The image is processed by subtracting and making the image binary, which indicates the movement of the wheelchair. In addition, ref. [80] features a wheelchair transformation system developed in SimWise, Simulink, and MATLAB^®^ scripts. The system implements a fuzzy logic controller. Ohtsuka et al. [81] describe a method that uses laser sensors and a joystick in order to control the position of the wheelchair. Finally, a virtual environment was developed by Qassim and Lakany [36], where a map is created through laser sensors for training and executing actions in case obstacles are found.

## 4. Discussion

The review of the literature is focused on the research into the instrumentation and control systems of wheelchairs. The development of the control systems taken into account for the study was carried out between 2012 and 2019. The two categories classify the instrumentation applied to the wheelchair and its user. The review includes 92 articles, two postgraduate theses, one book and two review articles regarding wheelchair control. 

The book mentions the devices for the mobility of and manipulation for people with reduced capacities, mentioning assistive tools, such as wheelchairs and walkers, describing the interfaces that use EEG, EOG, video-oculography (VOG), EMG, pressure signals and head movements through IMU [65]. Additionally, in [100] a survey is conducted on the topic of human-machine interactions for the control of a wheelchair. It also designs an interface based on facial expressions, which implements Viola Jones’ algorithm for the detection of faces. Another algorithm is used for the detection of the eyes and blinking. Concerning the systematic review carried out [101], the book broadens the outlook with regard to the control systems implemented between 2004 and 2016, as expressed by the authors, to continue expanding the review on the topic. Consequently, the review indicates that from the categories arise multiple systems and that the authors make an effort to solve daily problems that people with physical disabilities are faced with. The control methods are numerous and it is not possible to define one specific type of control for each area or one that depends directly on the type of sensors used, without excluding that every control system should have a model or transfer function which defines the input and output characteristics of the system for the correct execution of the controller. 

Based on the revision presented in this article, it is observed that the most frequent input methods used were: EEG signals through the use of a BCI interface, with 21 articles that presented this type of design, followed by 13 articles that used video cameras to control distance or capture the movement of the body parts of the user to operate the wheelchair. There were 12 articles that used controllers based on the head movement of the user, mostly benefitting users with disabilities in upper and lower limbs. Also, 10 articles used voice recognition systems and 10 articles used or improved a joystick for people with a disability in the lower limbs. In addition, the controllers most used to operate wheelchairs were obstacle detection algorithms, reported in 32 articles. Also, 11 articles implemented position controllers and eight velocity controllers. Regarding voice command controllers, there were eight articles and 11 that used controllers through signal thresholding.

### 4.1. Discussion on Instrumentation Non-Invasively Placed on the User 

Non-invasive methods refer to the instrumentation placed on the user’s body for the control of the wheelchair. The cost is low, but it depends on the technique used for capturing body signals. For example, BCI systems require electrodes for the capturing of EEG signals, which are expensive and the extraction is complex as, due to their nature, they require the filtering and amplification of the signals. However, they are very useful for people who suffer from a disability in their lower and upper limbs.

For their part, MEMS are easy to place on the user’s body due to their physical characteristics, however, the control system should have special care with the calibrations of said sensors. There are 8 articles related to MEMS, but they are less frequently found in comparison to articles that implement BCI. The reason for this may be the increase, in recent years, in the number of interfaces which interact with the person through sensors non-invasively placed on the user’s body.

Concerning the types of sensors which extract body characteristics, those that collect EOG signals can be mentioned. They are useful for people with no mobility in their lower and upper limbs. In this type of systems, it is important to define if they are active or not, given that the user should know when they are operating the system using body movements and when a natural action which does not interfere with the control of the wheelchair is being executed. These signals also present the characteristic of previous conditioning due to their nature.

Voice recognition systems are based on commands recognised by the controller and that are associated with a control action of the wheelchair. Nonetheless, they should be activated though an interface, in order to know if the controller is active or not. 

The types of HMI should be separated from other types of control because of the interaction they have with the user. Thus, HMI normally presents a system which is inherent to the sense organs of the person. For this, the graphic interfaces which are described in these articles should be designed for users with specific characteristics, given that not all wheelchairs are generic, that is, each designer develops their system thinking about the needs of a type of user and the pathologies or disabilities they have.

In the same way, the design of controllers not only focuses on the mobility of the wheelchair but also on the user’s comfort, the situations of risk they must be faced with, and the person’s environment. Said controllers can be suitable, depending on the characteristics of the user, the processing characteristics and the elements used to execute the controller, and the cost of the designs. Below, a comparison is presented of the characteristics mentioned in the articles with the more frequent control strategies that were dealt with in this investigation.

#### 4.1.1. Characteristics of the User

The designs created for specific users guarantee the full functioning of the controller, as well as the prior training to operate the wheelchair through thoughts or emotions. However, the manufacturing of generic controllers may present flaws, as indicated in Ng et al. [16] and Carrillo et al. [17], where the participation of 60 individuals indicates that the control did not work for most of the participants. Conversely, concerning MEMS sensors, the characteristics of the user are not as important as they are in the case of BCI, given that the sensors acquire movement signals which any user can generate. Yet, the user does require previous training to learn about the angles of movement of the head or hands. In the case of sEMG and EOG, as reported in some articles in this review, the characteristics of the user were not mentioned as the testing of the controller with a considerable number of participants is not described. Still, the physical characteristics of the user can be a determining factor for the implementation of these methods. 

#### 4.1.2. Processing Characteristics and Design Computational Cost

The computational cost is high when the controllers involve processing algorithms for EEG signals, as indicated in [7,8,9,10,11,12,15,16,17,21,22] and robust controls such as fuzzy logic and adaptive controllers, and the use of neural networks [6,11,18,23,25]. The computational cost is reduced when classic controllers are implemented, as is the case of the designs in [6,10,14,15,25]. With regard to controllers which involve MEMS or cameras to detect movement, ref. [52,56,58,61] have a higher computational cost, due to the implementation of algorithms and neural networks, but the controllers based on thresholds present in the MEMS category have a lower computational cost. The computational expense in controllers which involve sEMG and EOG was higher in articles [29,69,72]. The rest of the controllers involve thresholds for the control and use of PID controllers.

As per the above, the controllers developed with input methods based on the user depend on the user’s characteristics and the degree of mobility of the lower and/or upper limbs. They can also be implemented depending on the tools the designer has, such as the type of processor or microcontroller and the processing capacity. This review presents a wide variety of methods, algorithms, classic and robust controllers. The technological development has involved the use of artificial intelligence to predict behaviour, teach the system to make decisions through the use of neural networks, among others. 

### 4.2. Discussion on Wheelchair Instrumentation 

Obstacle detection and collision avoidance systems constitute a fundamental element in the design of a controller for a wheelchair, given that what is most important is the life and good health of the user. Hence, the system should always protect the person using the wheelchair. Detection systems are based on the use of sensors that measure distance or that receive signals from the external world about the presence of unknown objects. For this reason, this classification is made, knowing that the authors not only use sensors as a preventive measure but also as reference signals for the control of the wheelchairs. Artificial vision is an area openly explored in recent years, however, it requires considerable computational costs due to the resources used for the processing of the images. Additionally, the user needs this processing to be carried out in real time. For this, the use of FPGA is more common because it conducts parallel tasks which make processes significantly more agile. Although an FPGA can solve the problem, sometimes a more robust processor is required and, therefore, some of the systems described in this area carry a computer for its execution.

Finally, navigation systems give a general view about controllers which use communication protocols for the sending and receiving of data from sensors and actuators. They also improve the commercial systems of wheelchairs with a joystick or with tracking-systems for people and objects.

The articles presented in the area of instrumentation incorporated in wheelchairs mention important characteristics in the following manner: 

#### 4.2.1. Wheelchair Instrumentation and HMI

The sensors included in this revision present information about wheelchairs, however, one of the principal motives is to guarantee the safety of the user. To this end, ref. [6,10,11,14,16,18,20,23,32,33,36,38,40,42,43,44,50,58,59,60,64,67,71,74,75,76,77,81,87,88] include safety systems to avoid obstacles. This indicates that approximately 40% of the articles mention the use of algorithms with ultrasonic sensors, lasers, infrared, cameras, among others, in order to provide security in the movement of wheelchairs and avoid crashes. Nevertheless, those articles use another type of instrumentation to make their control more robust. Also, there are few studies that deal with the detection of obstacles, as it would require other types of input to control the wheelchair. 

#### 4.2.2. Computational Cost

The controllers that process images have a high computational cost, and it is for this reason that they are developed in computers and the use of microcontrollers or small processors is not implemented. In the case of [44,87,94], image processing is carried out using FPGA, which includes a number of necessary resources and components for those designs. In the case of image processing in computers, it is difficult to execute in real time and the data processing is slow due to the sequentiality of the software. The controllers that use a classic control can be applied in smaller devices and do not require prior training. Also, it is highlighted that the communication systems are faster if they implement nodes, GSM networks that are not that much used at present and also Bluetooth modules.

Based on the analysis carried out previously, the controllers that use HMI are useful for people with disabilities in the lower and upper limbs, while those systems that use wheelchair instrumentation are mostly useful for people with little movement in their lower limbs. The controllers are efficient, depending on the instrumentation and the processing that they use. However, the controllers designed should have a safety system for the user, as any fall or crash could put the life of the user at risk.

Within the expectations for the future, a design of controllers is hoped for that will offer safety in the mobility of wheelchair users, a high degree of precision in wheelchair instrumentation and low costs, making use of the technologies and methods described in the reviewed literature. Also, the exploration and implementation of controls in open spaces is required, as the majority of those described in the literature were tested in closed environments or in laboratories. 

## 5. Conclusions

The control systems developed between 2012 and 2019 include implementations which are non-invasive for the user, delving into BCI, given that there are more works related to this topic. Furthermore, HMI makes it more interactive and allows the system to relate to the user’s everyday life. In addition, artificial intelligence is contributing to this field by developing interfaces that permit the controlled system to make decisions regarding the person’s mobility. This work also contributes with the number of participants who validate the instrumented and controlled system of the completed wheelchair or prototype.

Taking into consideration that this article involves 97 documents in the range of dates established, the analysis indicates that 40% of the articles use security systems to avoid obstacles, this being a percentage that does not guarantee the safety of the patient. Besides, the most commonly used techniques involve EEG signals, head movements, the use of cameras, controls through voice patterns, AI techniques, classic controls, and the use of thresholds to determine actions in the motors of the wheelchair. Among the techniques most frequently used, controls of position, velocity and acceleration can be found, in addition to thresholds to determine actions in the motors of the wheelchair. The authors also involve the use of neural networks so the system learns based on input data. The selection of a certain controller cannot be suggested given to the diversity of designs presented in this review, which are part of different types of instrumentations. Notwithstanding, it can be affirmed that the instrumentation with a lesser margin of error makes for a better design of controller in any area of control theory. This article indicates that the techniques that can be implemented in users with disabilities in their lower and upper limbs are EEG, sEMG, and EOG, and the rest of the techniques mentioned are useful for people with disabilities in their lower limbs only. 

From the point of view of the investigation, the controllers that have been developed lately involve the use of EEG, head movement, voice commands, among others. However, not all of them have security systems and they are mainly very expensive to manufacture. For this reason, building a controller that involves a robust security system based on head movements using IMU is a challenge. The implementation of these designs can improve the quality of life of people with disabilities in lower and/or upper limbs and can be used in different conditions or spaces in order to guarantee their adequate functioning and the mobility of the user. 

## Figures and Tables

**Table 1:** Input methods of the controllers in the articles reviewed.

Method	References
Brain-computer interface (BCI)	[6,7,8,9,10,11,12,13,14,15,16,17,18,19,20,21,22,23,24,25,26]
Artificial vision	[6,11,14,27,28,29,30,31,32,33,34,35,36]
Voice commands	[37,38,39,40,41,42,43,44,45,46]
Touch	[47,48,49,50,51]
Hand movement	[52,53,54,55,56]
Head movement	[27,28,37,57,58,59,60,61,62,63,64,65]
EOG	[29,30,31,66,67]
sEMG	[68,69,70,71,72,73]
Avoid obstacles	[32,33,74,75,76,77]
Laser	[25,33,63,78,79,80,81]
Manual control/joystick	[21,47,49,62,79,82,83,84,85,86]

**Table 2:** Control strategies.

Control Strategy	References
Algorithm to avoid obstacles	[6,10,11,14,16,18,20,23,32,33,36,38,40,42,43,44,50,58,59,60,64,67,71,74,75,76,77,81,87,88]
Adaptive control	[6,18,52,53,82]
Motor Imagery and P300 potential	[7,8,10,15]
Emotion Fractal Analysis Method (EFAM)	[9]
Neural networks	[11,25,27,30,59,61,72,84]
Linear Discriminant Analysis (LDA)	[12]
Support Vector Machine (SVM)	[13]
Position control	[14,31,34,36,47,48,49,66,68,73,79]
Steady State Visually Evoked Potential (SSVEP)	[16,17,25]
Multi-Modal Control	[19]
eSense^TM^ algorithm	[21]
Classification method using spatial-frequency feature of steady- state somatosensory evoked potentials (SSSEPs)	[22]
Adaptive neuro-fuzzy inference system (ANFIS)	[23]
Adaboost Algorithm/Image Processing	[23,27,29,33,34,35,44,50,78,89]
Bayes method	[52]
Orientation control/Thresholding	[21,25,37,53,54,56,59,60,62,64,68]
PID/PI/PD Controller	[54,55,67,80,90,91,92]
Hilbert Algorithm	[56]
Velocity control	[32,34,69,70,71,74,82,83]
Voice control	[38,39,40,41,42,43,46,88]
Fuzzy logic control	[45,64,76,86,90,93]
Kalman Filter	[64,77]
Navigation control	[94]
Speeded-Up Robust Features (SURF) algorithm	[94,95]
Tracking control	[6,85,96]

**Table 3:** Number of participants per category.

**Instrumentation Non-Invasively Placed on the User**	
Sub-category	Participants
Brain-Computer Interface (BCI)	150
Systems which implement micro- electromechanical sensors (MEMS)	13
Surface Electromyography (sEMG) and Electro-oculography (EOG)	16
Other types of instrumentation	25
Total	204
**Instrumentation Incorporated in the Wheelchair**	
Sub-category	Participants
Obstacle detection	5
Artificial vision	3
Wheelchair navigation	42
Total	50

**Table 4:** Instrumentation and control strategies for BCI systems.

Instrumentation	Control Strategy	References
EEG headset, camera.	Finite state machine, wall tracking algorithm and obstacle avoidance. Adaptive control.	[6]
EEG headset (patterns)	Pattern extraction and signal classification.	[7]
EEG headset, Arduino, module Bluetooth HC-05.	Signal treatment through spectrum analysis.	[8]
EEG headset, computer.	Emotion Fractal Analysis Method (EFAM).	[9]
Smartphone, EEG headset, IR camera, and microcontroller.	The processing of EEG signals is carried out in a mobile phone and the IR camera detects obstacles.	[10]
EEG headset, Arduino, Raspberry Pi, camera, ultrasonic and LiDAR sensors, GPS module.	Signal processing and classification through convolusional neuronal networks (CNN).	[11]
EEG headset, computer, network module, motor control module.	Signal processing of through visual stimuli using linear discriminant analysis (LDA).	[12]
EEG headset, driver and motors.	Processing through Wavelet and Support Vector Machine (SVM) as classification method.	[13]
EEG headset, Bluetooth module, camera, computer, and wheelchair prototype.	Position control.	[14]
EEG headset, GPS module, computer, DC driver and motors.	Control through the processing of EEG signals and automated guided mode in LabVIEW^TM^.	[15]
EEG headset, laser and ultrasonic sensors, computer.	Steady State Visual Evoked Potential (SSVEP).	[16]
EEG headset, ultrasonic sensors, IMU, Arduino, LabVIEW^TM^.	Signal processing through SSVEP and gyroscope.	[17]
EEG headset, IMU, computer, wheelchair.	Controller elaborated with three operating modes, through EEG, IMU, and cognitive mode.	[18]
EEG headset, computer, HMI.	Adaptive algorithm with temporal oscillatory response.	[19]
EEG headset, sensors photoelectric sensors, Bluetooth module, computer.	Multi-modal control with extraction and pattern recognition.	[20]
EEG headset, computer, joystick.	Brain signals manipulate the joystick through eSense^TM^ algorithm.	[21]
EEG headset, microcontroller, computer, driver, and motors.	Classification method using spatial-frequency feature of steady- state somatosensory evoked potentials (SSSEPs). Filter and use of Fast Fourier Transform (FFT).	[22]
EEG headset, EMG, Raspicam, Raspberry, Arduino.	Use of adaptive neuro-fuzzy inference system (ANFIS) method and Adaboost algorithm.	[23]
EEG headset, computer, wheelchair.	Thought signals are processed and make the wheelchair work.	[24]
EEG headset, sEMG and EOG signals, virtual platform.	HMI permits wheelchair use simulatation using augmented reality (AR).	[25]
Ultrasonic sensors, computer.	Lyapunov method and adaptive control.	[26]

**Table 5:** Instrumentation and control strategies for MEMS technology.

Instrumentation	Control Systems	References
3-axis MEMS sensors, computer.	Bayes method and, linear and rotational control	[52]
Orientation and ultrasonic sensors, microcontroller.	Angle thresholding is carried out through Euler angles. PID control.	[53]
ADXL330, Rx and Tx system, computer.	Data reception and transmission system. It receives signals from the head and hands.	[54]
ADXL345, Arduino, XBee Series, Battery.	PID control to stabilize the system and maintain the range of angular values.	[55]
Piezoresistive sensores, 3D-MEMS sensors, WiFi module, computer, data acquisition card (DAQ).	The controller receives ECG signals from piezoresistive sensors, processed in LabVIEW^TM^ through the Hilbert algorithm.	[56]
Tilt sensor module, microprocessor, wheelchair.	The controller receives signals and a multiplexor selects them to operate the motors.	[57]
Capacitive sensors, computer.	Wavelet estimator and convolutional neural networks (CNN).	[58]
MEMS 3D sensors, ultrasonic, Bluetooth module, driver and motors.	Data thresholding.	[59]
MPU6050, solar panel, Arduino, ultrasonic sensors.	Data thresholding.	[60]
IMU, Arduino, computer.	Euclidean and Mahalanobis distance classifier. Artificial neural networks.	[61]
Infrared and inertial sensors, joystick, microcontroller, WiFi module.	Data thresholding.	[62]
Orientation sensors, voice recognition module, microcontroller, and LCD.	Orientation control and voice.	[37]
Stereo camera, GUI, computer.	Finite state machine.	[27]
Camera, motion sensors, computer, wheelchair.	Orientation control	[28]
Laser sensor, Kinect, and sEMG signals, computer and wheelchair.	PID control and processing of sEMG signals.	[63]

**Table 6:** Instrumentation and control strategies implemented for signals or body movements.

Instrumentation	Control Systems	References
Infrared and ultrasonic sensors, microcontroller.	Infrared sensors are placed on a pair of glasses the patient is wearing and if they open or close their eyes, in a certain order, this executes an action in the wheelchair.	[66]
EOG electrodes, RF transmitter and receiver, Encoder, Decoder, ultrasonic sensors.	PID control and EOG signal processing.	[67]
Video camera, computer.	Wavelet transform and, Adaboost and Haar algorithm. Control through neural networks.	[29]
Video camera, controller.	Image processing using Hough transformation.	[30]
Camera, Raspberry Pi, computer, arduino.	Image processing on a bidimensional plane.	[31]
sEMG signals, controller, wheelchair.	Processing and thresholding signals.	[68]
EMG signals, encoder, wheelchair.	The signals are processed through the square root and normalisation. Angular and linear velocity control.	[69]
sEMG signals, computer, wheelchair.	Signal normalisation and, angular and linear velocity control.	[70]
Kinect sensor and sEMG signals, wheelchair.	sEMG signals control the wheelchair using hand movements and the Kinect sensor is a security system.	[71]
IMU, computer.	Neural networks to control the wheelchair and use of IMU.	[72]
sEMG signals, computer, virtual wheelchair.	Spectrum addition to process sEMG signals and a peak detector.	[73]

**Table 7:** Other types of instrumentation and control strategies.

Instrumentation	Control Systems	References
Wheelchair, motors, ascending-descending stairs system.	Modular Fuzzy control using PD-fuzzy logic controller.	[90]
EOG and EEG signals, computer, wheelchair.	Voice recognition and BCI through signal and visual image processing. Simultaneous localization and mapping (monocular SLAM) algorithm.	[38]
EEG headset, Arduino, Bluetooth module, voice module.	The interface permits the selection of the operation mode of the chair and the signals are processed in the program.	[39]
Microphone, microcontroller, driver, motors.	Voice command control.	[40]
voice module, conditioning, and motors.	Voice control and key control	[41]
Joystick, PLC, smartphone, router, wheelchair.	The system receives signals from a screen or joystick, and they are processed and received by the PLC, taking into account changes in direction and rotation	[47]
GSM module, ultrasonic sensors, WiFi module, touch screen, prototype.	Position control which avoids obstacles.	[48]
Voice module, ultrasonic sensors, Arduino.	Voice command control to direct the chair. Security control.	[42]
Voice module, GPS module, IR sensors, prototype.	Voice command control in the speaker dependent mode.	[43]
Joystick, computer, wheelchair.	Neural networks and an interface to process information.	[49]
Joystick, encoder, microcontroller, wheelchair.	Adaptive velocity control.	[82]
Joystick, driver, motors, prototype.	Velocity control.	[83]
Computer, actuators, wheelchair.	Position and velocity control.	[97]

**Table 8:** Instrumentation and control strategies for obstacle detection.

Instrumentation	Control Systems	References
Ultrasonic sensors, computer, wheelchair.	Control to avoid obstacles through odometry. Speed control.	[74]
Camera, GPS module, computer, wheelchair.	Rotational velocity control and use of nodes and databases to operate the wheelchair.	[32]
Laser sensor, GPS module, accelerometer, Arduino.	Barrier detector system analysing the rotation of the wheels.	[75]
Ultrasonic sensors, encoder, computer.	Fuzzy logic control to avoid obstacles.	[76]
Laser camera, computer.	Conic coefficients for the processing of images and determining of distance	[33]
Ultrasonic sensors, encoder.	Extended Kalman filter for sensor signal treatment.	[77]

**Table 9:** Instrumentation and control strategies for artificial vision.

Instrumentation	Control Systems	References
Video camera, FPGA.	SURF detector. Computer vision algorithms.	[94]
FPGA, wheelchair.	Navigation control.	[87]
Voice module, IR sensors, LabVIEW^TM^, FPGA.	Voice control commands to activate the chair and detect obstacles through IR sensors.	[44]
Kinect, computer, wheelchair.	Triangulation method, proximity, motion recognition, gesture recognition and scene analysis. Position control.	[88]
PixyCMUcam5, ultrasonic sensors, microcontroller, transaxle motor.	Colour tracking technique. Image processing and obstacle detection system.	[98]
Pan-Tilt-Zoom (PTZ) c, gyroscope, encoder, laser, wheelchair.	Extended Kalman Filter, SURF algorithm. Fuzzy logic control and obstacle avoidance.	[95]
Webcam, ultrasonic sensors, DSP processor, computer.	Adaboost algorithm. Thresholding control.	[64]
Touch screen, DAQ, encoder, wheelchair.	PID control for rotational velocity and position.	[50]
Camera, computer, wheelchair.	Simultaneous Localization and Mapping (SLAM). Object segmentation and detection.	[34]
Stereo camera, computer, wheelchair.	Image processing to follow the legs of a companion.	[78]

**Table 10:** Instrumentation and control strategies for wheelchair navigation.

Instrumentation	Control Systems	References
Joystick, laser sensor, wheelchair.	Line control tracking.	[84]
		
Computer, wheelchair.	Map estimation of the companion’s position	[85]
		
GSM module, WiFi module, IR sensors, Arduino, wheelchair.	Control through touch and security screen.	[89]
Computer, wheelchair.	PI motion control.	[86]
Computer, wheelchair.	Tracking control and wheelchair rotation.	[99]
Trackball sensor, ultrasonic sensors, computer, wheelchair.	PID motion control.	[93]
Voice modules, ultrasonic sensors, microcontroller, LCD, prototype.	Voice control through commands.	[51]
		
Laser sensors, computer, LabVIEW^TM^.	Locomotion and posture control. PI and PD control.	[91]
Camera, ultrasonic sensors, computer, wheelchair, bed.	Position control and obstacle avoidance.	[96]
Joystick, computer, wheelchair	Neural networks and LabVIEW^TM^.	[92]
Camera, computer, wheelchair.	HAAR Cascade Algorithm, image processing and path planning.	[35]
Joystick, computer, wheelchair.	Vector Field Histogram (VHF) algorithm, fuzzy logic control in LabVIEW^TM^.	[45]
Computer, wheelchair.	Predictive tracking model and transverse feedback linearization.	[46]
LED matrix, cameras.	Image processing and classification.	[79]
Computer, wheelchair.	Fuzzy logic control.	[80]
Laser sensors and joystick, computer, wheelchair.	Position control.	[81]
Laser sensors, computer, wheelchair.	Virtual environment elaborated in MATLAB^®^ to execute actions in case of obstacles.	[36]

## References

[1] World Health Organization International Classification of Functioning, Disability and Health (ICF). https://www.who.int/classifications/icf/en/.

[2] World Health Organization Fact Sheet on Wheelchairs. https://apps.who.int/iris/handle/10665/205041.

[3] Sala Situacional de Personas con Discapacidad. https://www.minsalud.gov.co/sites/rid/Lists/BibliotecaDigital/RIDE/VS/MET/sala-situacional-discapacidad2019-2-vf.pdf.

[4] World Health Organization World Report on Disability. https://www.who.int/disabilities/world_report/2011/report/en/.

[5] Información Estadística de la Discapacidad. https://www.dane.gov.co/files/investigaciones/discapacidad/inform_estad.pdf.

[6] Widyotriatmo A., Suprijanto, Andronicus S. A collaborative control of brain computer interface and robotic wheelchair. Proceedings of the 10th Asian Control Conference (ASCC 2015).

[7] Long J., Li Y., Wang H., Yu T., Pan J., Li F. (2012). A Hybrid Brain Computer Interface to Control the Direction and Speed of a Simulated or Real Wheelchair. IEEE Trans. Neural Syst. Rehabil. Eng..

[8] Dabosmita P., Moumita P. Automation of wheelchair using brain computer interface (BCI) technique. Proceedings of the American Institute of Physics (AIP 2019).

[9] Kobayashi N., Nakagawa M. BCI-based control of electric wheelchair. Proceedings of the 4th Global Conference on Consumer Electronics (GCCE 2015).

[10] Jayabhavani G., Raajan N., Rubini R. Brain mobile interfacing (BMI) system embedded with wheelchair. Proceedings of the IEEE Conference on Information and Communication Technologies (ICT 2013).

[11] Zgallai W., Brown J., Ibrahim A., Mahmood F., Mohammad K., Khalfan M., Mohammed M., Salem M., Hamood N. Deep Learning AI Application to an EEG driven BCI Smart Wheelchair. Proceedings of the Advances in Science and Engineering Technology International Conferences (ASET 2019).

[12] Kim K., Carlson T., Lee S. Design of a robotic wheelchair with a motor imagery based brain-computer interface. Proceedings of the International Winter Workshop on Brain-Computer Interface (IWW-BCI 2013).

[13] Reshmi G., Amal A. Design of a BCI system for piloting a wheelchair using five class MI Based EEG. Proceedings of the 3rd International Conference on Advances in Computing and Communications (ICACC 2013).

[14] Jang W., Lee S., Lee D. Development BCI for individuals with severely disability using EMOTIV EEG headset and robot. Proceedings of the International Winter Workshop on Brain-Computer Interface (IWW-BCI 2014).

[15] Huang C., Wang Z., Chen G., Yang C. Development of a smart wheelchair with dual functions: Real-time control and automated guide. Proceedings of the 2nd International Conference on Control and Robotics Enginnering (ICCRE 2017).

[16] Ng D., Soh Y., Goh S. Development of an autonomous BCI wheelchair. Proceedings of the Symposium on Computational Intelligence in Brain Computer Interfaces (CIBCI 2104).

[17] Carrillo C. (2017). Diseño y Construcción de una Interfaz Cerebro Computadora Para el Control de una Silla de Ruedas Como Ayuda a Personas con Discapacidad Motriz. Master’s Thesis.

[18] Lasluisa N. (2016). Diseño y Construcción de una Silla de Ruedas Autónoma Mediante Ondas Cerebrales. Bachelor’s Thesis.

[19] Pinos E., Guevara D., López F. Electroencephalographic signals acquisition for the movement of a wheelchair prototype in a BCI system. Proceedings of the Asia-Pacific Conference on Computer Aided System Engineering (APCASE 2015).

[20] Chen N., Wang X., Men X. Hybrid BCI based control strategy of the intelligent wheelchair manipulator system. Proceedings of the Conference on Industrial Electronics and Applications (ICIEA 2018).

[21] Su Z., Xu X., Ding J., Lu W. Intelligent wheelchair control system based on BCI and the image display of EEG. Proceedings of the IEEE Advanced Information Management, Communicates, Electronic an Automation Control Conference (IMCEC 2016).

[22] Kim K., Lee S. Steady-state somatosensory evoked potentials for brain-controlled wheelchair. Proceedings of the International Winter Workshop on Brain-Computer Interface (IWW-BCI 2014).

[23] Turnip A., Hidayat T., Kusumandari D. Development of brain-controlled wheelchair supported by raspicam image processing based Raspberry Pi. Proceedings of the 2nd International Conference on Automation, Cognitive Science, Optics, Micro Electro-Mechanical Systems and Information Technology (ICACOMIT 2017).

[24] Shahin M., Tharwat A., Gaber T. (2019). A Wheelchair Control System Using Human-Machine Interaction: Single-Modal and Multimodal Approaches. J. Intell. Syst..

[25] Borges L., Martins F., Naves E., Bastos T., Lucena V. Multimodal system for training at distance in a virtual or augmented reality environment for users of electric-powered wheelchairs. Proceedings of the International Federation of Automatic Control (IFAC).

[26] Chrisander S., Widyotriatmo A. Wall following control for the application of a brain-controlled wheelchair. Proceedings of the International Conference on Intelligence Autonomous Agents, Networks and Systems (INAGENTSYS 2014).

[27] Yoda I., Tanaka J., Raytchev B., Sakakue K., Inoue T. Stereo camera based non-contact non-constraining head gesture interface for electric wheelchairs. Proceedings of the 18th International Conference on Pattern Recognition (ICPR’06).

[28] Manta L., Cojocaru D., Vladu I., Dragomir A., Marin A. Wheelchair control by head motion using a noncontact method in relation to the pacient. Proceedings of the 20th International Carpathian Control Conference (ICCC 2019).

[29] Pangestu G., Utaminingrum F., Bachtiar F. (2019). Eye state recognition using multiple methods for applied to control smart wheelchair. Int. J. Intell. Eng. Syst..

[30] Rajesh A., Mantur M. Eyeball gesture controlled automatic wheelchair using deep learning. Proceedings of the Region 10 Humanitarian Technology Conference (R10-HTC 2017).

[31] Wanluk N., Visitsattapongse S., Juhong A., Pintavirooj C. Smart wheelchair based on eye tracking. Proceedings of the 9th Biomedical Engineering International Conference (BMEiCON 2016).

[32] Sumida Y., Hayashi M., Goshi K., Matsunaga K. Development of a route finding system for manual wheelchair users based on actual measurement data. Proceedings of the 9th International Conference Ubiquitous Intelligence and Computing and 9th International Conference on Autonomic and Trusted Computing (UIC-ATC 2012).

[33] Natraj A., Natraj S., Waharte S., Kroening D. Camera-laser projector stereo system based anti-collision system for robotic wheelchair users with cognitive impairment. Proceedings of the International Conference on Multisensor Fusion and Information Integration for Intelligent Systems (MFI).

[34] Couceiro M. (2019). Wheelchair Navigation: Automatically Adapting to Evolving Environments. Towards Auton. Robot. Syst..

[35] Puanhvuan D., Khemmachotikun S., Wechakam P., Wijam B., Wongsawat Y. Automated navigation system for eye-based wheelchair controls. Proceedings of the Biomedical Engineering International Conference (BMEiCON 2014).

[36] Qassim H., Lakany H. Virtual environment modelling using simulated laser scanners. Proceedings of the 2nd International Conference on Electrical, Communication, Computer, Power and Control Engineering (ICECCPCE).

[37] Ruzaij M., Neubert S., Stoll N., Thurov K. Multi-sensor Robotic-wheelchair controller for Handicap and Quadriplegia patients using embedded technologies. Proceedings of the International Conference on Human System Interactions (HIS 2016).

[38] Karpov V., Malakhov D., Moscovsky A., Robvo M., Sorokoumov P., Velichskovsky B., Ushakov V. (2019). Architecture of a wheelchair control system for disabled people: Towards multifunctional robotic solution with neurobiological interfaces. Sovrem. Technol. Med..

[39] Boucha D., Amiri A., Chogueur D. Controlling electronic devices remotely by voice and brain waves. Proceedings of the International Conference on Mathematics and Information Technology (ICMIT 2017).

[40] Ruzaij M., Poonguzhali S. Design and implementation of low cost intelligent wheelchair. Proceedings of the International Conference on Recent Trends Information Technology (ICRTIT 2012).

[41] Wang D., Yu H. Development of the control system of a voice-operated wheelchair with multi-posture characteristics. Proceedings of the 2nd Asia-Pacific Conference on Intelligent Robot Systems (ACIRS 2017).

[42] Umchid S., Limhaprasert P., Chumsoongnern S., Petthong T., Leedomwong T. Voice controlled automatic wheelchair. Proceedings of the Biomedical Engineering International Conference (BMEiCON 2018).

[43] Aktar N., Jaharr I., Lala B. Voice recognition based intelligent wheelchair and GPS tracking system. Proceedings of the 2nd International Conference on Electrical, Computer and Communication Engineeering (ECCE 2019).

[44] Kathirvelan J., Anilkumar R., Alex Z., Fazul A. Development of low cost automatic wheelchair controlled by oral commands using standalone controlling system. Proceedings of the 2012 IEEE International Conference on Computational Intelligence and Computing Research (ICCIC 2012).

[45] Rabhi Y., Mrabet M., Fnaiech F., Gorce P. Intelligent joystick for controlling power wheelchair navigation. Proceedings of the 3rd International Conference on System Control (ICSC 2013).

[46] Nguyen V., Sentouh C., Pudlo P., Popieul J. Path following controller for electric power wheelchair using model predictive control and transverse feedback linearization. Proceedings of the International Conference on Systems, Man and Cybernetics (SMC 2018).

[47] Gulpanich S., Petchhan J., Wongvanich N. PLC-based wheelchair control with integration of the Internet of things. Proceedings of the 57th Annual Conference of the Society of Instrument and Control Engineers of Japan (SICE 2018).

[48] Makwana S., Tandon A. Touch screen based wireless multifunctional wheelchair using ARM and PIC microcontroller. Proceedings of the International Conference on Microelectronics, Computing and Communications (MicroCom 2016).

[49] Rabhi Y., Mrabet M., Fnaiech F. Optimized joystick control interface for electric powered wheelchairs. Proceedings of the 16th International Conference on Sciences and Techniques of Automatic Control and Computer Engineering (STA 2015).

[50] Solea R., Margarit A., Cernega C., Serbencu A. Head movement control of powered wheelchair. Proceedings of the 23rd International Conference on System Theory, Control and Computing (ICSTCC 2019).

[51] Sivakumar M., Murji J., Jacob L., Nyange F., Banupriya M. Speech controlled automatic wheelchair. Proceedings of the Pan African International Conference on Information Science, Computing and Telecommunications (PACT 2013).

[52] Lu T. A motion control method of intelligent wheelchair based on hand gesture recognition. Proceedings of the 8th Conference on Industrial Electronics and Applications (ICIEA 2013).

[53] Ruzaij M., Neubert S., Stoll N., Thurov K. Auto calibrated head orientation controller for robotic-wheelchair using MEMS sensors and embedded technologies. Proceedings of the Sensors Application Symposium (SAS 2016).

[54] Kaur S., Vashist H. (2013). Automation of Wheel Chair Using MEMS Accelerometer (ADXL330). Adv. Electron. Electr. Eng..

[55] Canagareddy D., Subarayadu K., Hurbungs V. Hand gesture controller for robotic-wheelchair using microelectromechanical sensor ADXL 345. Proceedings of the International Conference on Emerging Trends in Electrical, Electronic and Communications Engineering (ELECOM 2018).

[56] Postolache O., Viegas V., Dias J., Vinhas J., Silva P., Postolache G. Toward developing a smart wheelchair for user physiological stress and physical activity monitoring. Proceedings of the International Symposium on Medical Measurements and Applications (MeMeA 2014).

[57] Chen Y., Chen S., Chen W., Lin J. (2003). A head orientated wheelchair for people with disabilities. Disabil. Rehabil..

[58] Dobrea M., Dobrea D., Severin I. A new wearable system for head gesture recognition designed to control an intelligent wheelchair. Proceedings of the E-Health and Bioengineering Conference.

[59] Kader M.A., Alam M.E., Jahan N., Bhuiyan M.A., Alam M.S., Sultana Z. Design and implementation of a head motion- controlled semi-autonomous wheelchair for quadriplegic patients based on 3-axis accelerometer. Proceedings of the 22nd International Conference on Computer and Information Technology (ICCIT 2019).

[60] Dey P., Hasan M., Mostofa S., Rana A. Smart wheelchair integrating head gesture navigation. Proceedings of the International Conference on Robotics, Electrical and Signal Processing Techniques (ICREST 2019).

[61] Marins G., Carvalho D., Marcato A., Junior I. Development of a control system for electric wheelchairs based on head movements. Proceedings of the Intelligent Systems Conference (IntelliSys 2017).

[62] Gomes D., Fernandes F., Castro E., Pires G. Head-movement interface for wheelchair driving based on inertial sensors. Proceedings of the Portuguese Meeting on Bioengineering (ENBENG 2019).

[63] Rohmer E., Pinheiro P., Cardozo E., Bellone M., Reina G. Laser based Driving Assistance for Smart Robotic Wheelchairs. Proceedings of the 20th Conference on Emerging Technologies & Factory Automation (ETFA).

[64] Jia P., Hu H. (2012). Head gesture recognition for hands-free control of an intelligent wheelchair. Ind. Robot.

[65] Bastos-Filho T., Kumar D., Arjunan S. (2014). Devices for Mobility and Manipulation for People with Reduced Abilities.

[66] Challagundla M., Yogeshwar K., Harsha N. Automatic motion control of powered wheelchair by the movements of eye blink. Proceedings of the International Conference on Advanced Communications, Control and Computing Technologies (ICACCCT 2014).

[67] Pingali T., Dubey S., Shivaprasad A., Varshney A., Ravishankar S., Pingali G., Polisetty N., Manjunath N., Padmaja K. Eye-gesture controlled intelligent wheelchair using Electro-Oculography. Proceedings of the International Symposium on Circuits and Systems (ISCAS 2014).

[68] Hardiansyah R., Ainurrohmah A., Aniroh Y., Tyas F. The electric wheelchair control using electromyography sensor of arm muscle. Proceedings of the International Conference on Information, Communication Technology and Systems (ICTS 2016).

[69] Jang G., Kim J., Lee S., Choi Y. (2016). EMG-Based Continuous Control Scheme with Simple Classifier for Electric-Powered Wheelchair. IEEE Trans. Ind. Electron..

[70] Jang G., Choi Y. EMG-based continuous control method for electric wheelchair. Proceedings of the International Conference on Intelligence Robots and Systems (IEEE/RSJ 2014).

[71] Küçükyildiz G., Ocak H., Şayli Ö., Karakaya S. Real time control of a wheelchair based on EMG and Kinect for the disabled people. Proceedings of the Medical Technologies National Conference (TIPTEKNO 2015).

[72] Fortune E., Cloud B., Madansingh S., Ngufor C., Straaten M., Goodwin M., Murphree D., Zhao K., Banitt M. (2019). Estimation of manual wheelchair-based activities in the free-living environment using a neural network model with inertial body-worn sensors. J. Electromyogr. Kines..

[73] Silva A., Morere Y., Naves E., Sa A., Soares A. Virtual electric wheelchair controlled by electromyographic signals. Proceedings of the Biosignals and Biorobotics Conference (ISSNIP BRC).

[74] Zhang J., Wang J., Chen W. A control system of driver assistance and human following for smart wheelchair. Proceedings of the International Conference on Robotics and Biomimetics (IEEE ROBIO 2014).

[75] Taniue H., Kaneko J., Kojima K. Development of automatic barrier detection system for wheelchair. Proceedings of the 4th Global Conference on Consumer Electronics (GCCE 2015).

[76] Lee Y., Chiu C., Kuo I. Fuzzy wall-following control of a wheelchair. Proceedings of the 17th World Congress of International Fuzzy Systems Association and 9th International Conference on Soft Computing and Intelligent Systems (IFSA-SCIS 2017).

[77] Maatoug K., Njah M., Jallouli M. Multisensor data fusion for electrical wheelchair localization using extended Kalman Filter. Proceedings of the 18th International Conference on Sciences and Techniques of Automatic Control and Computer Engineering (STA 2017).

[78] Motokucho T., Oda N. Vision-based human-following control using optical flow field for power assisted wheelchair. Proceedings of the International Workshop on Advanced Motion Control (AMC 2014).

[79] Tsunoda T., Premachandra C., Premachandra H. Visible light communication by using LED array for automatic wheelchair control in hospitals. Proceedings of the 23rd International Symposium on Consumer Technologies (ISCT 2019).

[80] Razali N., Ghani N., Jamin N., Masrom M. Stability control of wheelchair system using interval type-2 fuzzy logic control. Proceedings of the 9th IEEE Control System Graduate Research Colloquium (ICSGRC 2018).

[81] Ohtsuka H., Shibasato K., Shimada Y., Kato T. Hand-free maneuvering system for electric wheelchair using laser range finder. Proceedings of the 11th Asian Control Conference (ASCC).

[82] Clearesta E., Wardhana A.A., Widyotriatmo A., Suprijanto Adaptive control for velocity control of an electric wheelchair. Proceedings of the 3rd International Conference on Instrumentation, Control and Automation (ICA 2013).

[83] Matsuo K., Barolli L. Design and implementation of an omnidirectional wheelchair: Control system and its applications. Proceedings of the 9th International Conference on Broadband and Wireless-Computing, Communication and Applications (BWCCA 2014).

[84] Chen X., Agrawal S. (2013). Assisting versus repelling force-feedback for learning of a line following task in a wheelchair. IEEE Trans. Neural Syst. Rehabil. Eng..

[85] Sato Y., Suzuki R., Arai M., Kobayashi Y., Kuno Y., Fukushima M., Yamazaki K., Yamazaki A. Multiple robotic wheelchair system able to move with a companion using map information. Proceedings of the International Conference on Human-Robot Interaction (ACM/IEEE 2014).

[86] Feng G., Guerra T., Nguyen A., Busoniu L., Mohammad S. (2019). Robust Observer-Based Tracking Control Design for Power- Assisted Wheelchairs. IFAC-PapersOnLine.

[87] Megalingam R., Pillai M. FPGA based wheelchair autonavigation for people with mobility issues. Proceedings of the IEEE International WIE Conference on Electrical and Computer Engineering (WIECON-ECE 2015).

[88] Chang C., Chen C., Chen Y., Lin B. Kinect-based powered wheelchair control system. Proceedings of the International Conference on Intelligent Systems, Modelling and Simulation (ISMS 2013).

[89] Makwana S., Shah V., Mehta S. Prototype buildout of GUI based multifaceted automated wheelchair system. Proceedings of the International Conference on Intelligent Computing and Control Systems (ICICCS 2017).

[90] Tokhi M., Ghani N., Hassan M., Nasir A. A dual phase modular fuzzy control structure for an automode wheelchair in ascending and descending stairs. Proceedings of the Conference on Control and Automation.

[91] Chocoteco J., Morales R., Feliu V., Sánchez L. (2014). Trajectory Planning for a Stair-Climbing Mobility System Using Laser Distance Sensors. IEEE Syst. J..

[92] Rabhi Y., Mrabet M., Fnaiech F., Gorce P. A feedforward neural network wheelchair driving joystick. Proceedings of the International Conference on Electrical Engineering and Software Applications (ICEESA 2013).

[93] Sreejith T., Vishnu J., Vijayan G. Trackball controlled novel, cost effective electric wheelchair. Proceedings of the International Conference on Control, Power, Communication and Computing Technologies (ICCPCCT 2018).

[94] Zhao J., Huang X., Massoud Y. An efficient real-time FPGA implementation for object detection. Proceedings of the 12th International New Circuits and Systems Conference (NEWCAS 2014).

[95] Wu B., Jen C., Tsou T., Chen P. Accompanist recognition and tracking for intelligent wheelchairs. Proceedings of the International Conference on Systems, Man, and Cybernetics (SMC 2014).

[96] Liang X., Li X., Jia S., Sun Y. Wheelchair/bed docking control based on the combination of vision and ultrasound. Proceedings of the Chinese Automation Congress (CAC 2017).

[97] Cho B., Yoon J., Kho S., Lee J., Kwon S., An H., Lee S., Kim C. Seat posture stabilizing function for an electric wheelchair based on controlled pendulum mechanism. Proceedings of the 4th International Conference on Control, Decision and Information Technologies (CoDIT 2017).

[98] Ahmad M., Rong H., Alhady S., Rahiman W., Othman W. Colour tracking technique by using pixy CMUcam5 for wheelchair luggage follower. Proceedings of the 7th IEEE International Conference on Control System, Computing and Engineering (ICCSCE 2017).

[99] Park J., Im W., Kim D., Kim J. Safe driving algorithm of the electric wheelchair with model following control. Proceedings of the 16th European Conference on Power Electronics and Applications (EPE-ECCE 2014).

[100] Ghorbel A., Ben Amor N., Jallouli M. (2019). A survey on different human-machine interactions used for a survey on different human-machine interactions used for controlling an electric wheelchair. Procedia Comput. Sci..

[101] Wahyufitriyani C., Susmartini S., Priadythama I. Review of intelligent wheelchair technology control development in the last 12 years. Proceedings of the 2nd International Conference of Industrial, Mechanical, Electrical and Chemical Engineering (ICIMECE 2016).

